# The association between the MIND-NL diet, Dutch dietary guidelines, and global cognitive function in an older population at risk for cognitive decline

**DOI:** 10.1016/j.jnha.2025.100680

**Published:** 2025-09-08

**Authors:** Sonja Beers, Marian A.E. de van der Schueren, Pol Grootswagers, Ondine van de Rest, Lisa Waterink, Sietske A.M. Sikkes, Kay Deckers, Lion M. Soons, Jurgen A.H.R. Claassen, Nynke Smidt, Wiesje M. van der Flier, Sebastian Köhler, Esther Aarts, Yannick Vermeiren, Lisette CPGM de Groot, on behalf of MOCIA consortium FINGER-NL consortium

**Affiliations:** a Division of Human Nutrition and Health, Wageningen University & Research, Wageningen, The Netherlands; b Department of Nutrition, Dietetics and Lifestyle, HAN University of Applied Sciences, Nijmegen, The Netherlands; c Alzheimer Center Amsterdam, Neurology, Vrije Universiteit Amsterdam, Amsterdam UMC Location VUmc, Amsterdam, The Netherlands; d Amsterdam Neuroscience, Neurodegeneration, Amsterdam, The Netherlands; e Department of Psychiatry and Neuropsychology, Alzheimer Centrum Limburg, Mental Health and Neuroscience Research Institute (MHeNs), Maastricht University, Maastricht, The Netherlands; f Department of Geriatrics, Radboud University Medical Center, Radboudumc Alzheimer Center, Nijmegen, The Netherlands; g Radboud University, Donders Institute for Brain, Cognition and Behaviour, Nijmegen, The Netherlands; h Department of Cardiovascular Sciences, Leicester University, Leicester, United Kingdom; i Department of Epidemiology, University of Groningen, University Medical Center Groningen, Groningen, The Netherlands; j Epidemiology & Data Science, Vrije Universiteit Amsterdam, Amsterdam UMC Location VUmc, Amsterdam, The Netherlands

**Keywords:** Dietary pattern, Cognition, Network analysis, Elderly, Fruiting vegetables, Vitamin E

## Abstract

**Objectives:**

This study examined the association between adherence to the Dutch MIND diet (Mediterranean-DASH Intervention for Neurodegenerative Delay, MIND-NL) and the Dutch dietary guidelines (DHD2015-index) with global cognitive function in older adults at risk of cognitive decline.

**Design and setting:**

A cross-sectional study was conducted using baseline data of the FINGER-NL trial.

**Participants:**

A total of 1,135 older adults, aged 60–80 years, at risk for cognitive decline with complete dietary data and complete neuropsychological tests were included in the analyses.

**Measurements:**

A validated 72-item Food Frequency Questionnaire (FFQ) was used to assess adherence to the dietary patterns. Global cognitive function was assessed by calculating a composite score based on four subtests of a neuropsychological test battery. Multiple linear regression analyses, adjusted for age, sex, education level, socioeconomic status (SES), body mass index (BMI), physical activity level, smoking status, and cardiovascular risk factors, were applied to examine potential associations between MIND-NL diet score and global cognitive function, and between DHD2015-index and global cognitive function. Interaction and subsequent subgroup analyses were conducted based on age, sex, education, SES, and physical activity. Explorative network analyses were applied to identify links between individual dietary intake components and global cognitive function.

**Results:**

The median [IQR] age of the participants was 67 [64−71] years. Overall, neither the MIND-NL diet nor the DHD2015-index was associated with the global cognition composite score (β = 0.014, 95%CI: -0.016, 0.045, p = 0.35 and β = 0.003, 95%CI: -0.000, 0.006, p = 0.07, respectively). The association between MIND-NL diet score and global cognition was moderated by age (p_interaction_ = 0.06), with adults under 70 years of age showing a positive trend. Although no significant interaction was noted (p_interaction_ = 0.28), an association was found between DHD2015-index and global cognition in participants aged under 70 years (β = 0.004, 95%CI: 0.000, 0.008, p = 0.048). Dietary intake of fruiting vegetables and vitamin E were positively correlated with global cognitive function.

**Conclusion:**

In this study, adherence to the Dutch dietary guidelines was associated with better global cognitive function among older adults under the age of 70 years at risk of cognitive decline. Future research aims at investigating longitudinal associations and confirming the moderating effect of age.

## 1 Introduction

Evidence regarding the association between diet and cognitive ageing is inconsistent [1]. The Mediterranean-Dietary Approach to Stop Hypertension (DASH) Intervention for Neurogenerative Delay (MIND) diet has emerged as a promising dietary approach to reduce cognitive decline and dementia risk [2,3]. However, van Soest et al. and Shakersain et al. proposed that the association between the original U.S.-developed MIND diet and brain ageing may differ in populations with other food cultures [4,5]. The original U.S.-developed MIND diet is a food-based dietary pattern. However, dietary habits can differ between countries or even between ethnic groups within a country. When applying the MIND diet across cultures, local dietary habits may require adaptation of food groups, which may consist of different food products across countries. This asks for cultural adaptation of the original U.S.-developed MIND diet.

To date, evidence on the association between the MIND diet and cognitive functioning is inconclusive. A review including six cross-sectional studies supported positive associations between the MIND diet and global cognitive functioning and episodic memory, whereby most findings originated from North American populations [4]. Furthermore, it is unknown whether MIND diet is stronger associated with cognitive function compared to national dietary guidelines or other well-established dietary patterns (e.g. DASH and Mediterranean diets). Prospectively, in an American cohort it was reported that the MIND diet was stronger associated with a reduction in cognitive decline and Alzheimer's disease than both the DASH and Mediterranean diets [2,3]. Australian prospective research assessing both adherence to national dietary guidelines and MIND diet in relation to cognitive impairment, also concluded there is added value to the MIND diet [6]. However, a Swedish and Dutch cohort did not directly confirm stronger associations for the MIND diet compared to national dietary guidelines for cognitive decline and dementia incidence, respectively [5,7].

The current Dutch dietary guidelines, outlined in the Dutch Healthy Dietary Index 2015 (DHD2015-index), are based on former evidence for the prevention of ten major chronic diseases. Although dementia is one of these ten major chronic, noncommunicable diseases, the committee stated that there was only sufficient evidence available for reduced alcohol intake and dementia risk reduction [8]. There is little evidence on the association between the Dutch dietary guidelines and cognition. A population-based cohort study assessing the association between the DHD2015-index and cognitive function reported that higher adherence to the Dutch dietary guidelines was associated with better cognitive performance at baseline and with less cognitive decline over a median follow-up of 15 years [9]. As yet, no other studies reported the association between the current Dutch dietary guidelines and cognitive functioning.

Previous studies of the MIND diet and DHD2015-index have been performed in the general older population, which is of interest for universal prevention [[2], [3], [4], [5], [6], [7],^9^]. However, evidence regarding at-risk populations for cognitive decline and dementia is scarce. Recent lifestyle interventions, including dietary advise, have shown that targeting individuals at increased risk of cognitive decline is most effective [10]. This underscores the further need to study the association of dietary patterns and cognitive functioning in at-risk populations.

Therefore, the current research aims to examine the potential association between adherence to the MIND-NL diet and the Dutch dietary guidelines (DHD2015-index) with global cognitive function in a Dutch population at risk for cognitive decline. Additionally, a data-driven approach was used to identify the most important food groups related to global cognitive function.

## 2 Methods

### 2.1 Study population

The present study made use of baseline data of the FINGER-NL study, a 2-year multicentre, multidomain lifestyle intervention in older adults (60–80 years old) at risk of cognitive decline (ID: NCT05256199) [11]. The FINGER-NL study was approved by the Medical Ethical Committee VU Medical Centre (NL77242.029.21; Amsterdam, The Netherlands) and conducted according to the Declaration of Helsinki. All participants provided written consent. Participants were recruited via an online recruitment platform Dutch Brain Research Registry (https://hersenonderzoek.nl/) [12]. In parallel, local participation pools and deployed initiatives assured additional recruitment.

Participants at risk for cognitive decline were screened for having at least one non-modifiable risk factor and at least two modifiable risk factors. Non-modifiable risk factors were defined as having a first-degree family history of dementia, or subjective memory complaints. Modifiable risk factors were defined as physical inactivity, unhealthy diet, low mental/cognitive activity, high blood pressure, high cholesterol, and high body mass index (BMI) (for 60–69 years old ≥25 kg/m^2^, for ≥70 years ≥28 kg/m^2^). Risk factors were all self-reported. Other inclusion criteria were sufficient knowledge of the Dutch language and having internet access at home to fill in online questionnaires. Exclusion criteria were self-reported diagnosis of dementia or mild cognitive impairment (MCI), cognitive impairment screened with the Modified Telephone Interview for Cognitive Status battery (TICSm score <23) [13], conditions affecting safe and continuous engagement in the intervention, major psychiatric disorders, symptomatic cardiovascular disease, re-vascularization within the last three months, and impaired vision, hearing, or mobility.

For the present study, we used baseline data to perform cross-sectional analyses. Baseline data were collected between January 2022 and June 2023 by five university research centres across the Netherlands: Amsterdam, Groningen, Maastricht, Nijmegen, and Wageningen. A total of 1210 participants were recruited for the study [11]. For the current study only participants with dietary data and complete neuropsychological tests were used for analyses.

### 2.2 Dietary assessment

Habitual dietary intake was assessed by a 72-item short FFQ, named the MIND-NL-Eetscore FFQ. Validation of this FFQ for both the MIND-NL score as well as DHD2015-index has previously been reported [14, Beers et al., unpublished results]. The FFQ assessed dietary intake of the past month. Participants were asked about the frequency of consuming food items, and how much per consumption they ate, based on standardized portion sizes and commonly used household measures. Furthermore, participants filled in three-day food records, from which average energy intake was estimated.

As a measure of the MIND diet, the MIND-NL diet score was calculated. The development of this score has been presented in a previous paper [15]. The MIND-NL diet comprises 15 food groups, of which nine are designated as healthy food groups (green leafy vegetables, other vegetables, berries and strawberries, nuts, beans and legumes, whole grains, poultry, fish, and olive oil) and six food groups are classified as unhealthy (red and processed meat, butter and stick margarines, full-fat cheese, take out, fried foods, and snacks, cookies, pastries, and sweets, and wine). For scoring of the food groups, pre-defined cut-off values were determined. Each food group can be scored 0.0, 0.5, and 1 with the exception of wine, which can be scored 0.0 or 1.0. The total MIND-NL score ranges between 0 and 15, with higher scores representing better adherence to the MIND-NL diet (Table 1).

**Table 1:** Dietary food groups and scoring for MIND-NL diet and DHD2015.

MIND-NL food groups	Score			DHD2015 food groups	Score	
	0	0.5	1		Minimum (0 points)	Maximum (10 points)
Green leafy vegetables	≤29 g/day	>29 - <100 g/day	≥100 g/day	Vegetables	0 g/d	≥200 g/d
Other vegetables	<71 g/day	≥ 71 -<100 g/day	≥100 g/day			
Berries and strawberries	<7 g/day	≥7 - <36 g/day	≥36 g/day	Fruit	0 g/d	≥200 g/d
Legumes	<9 g/day	≥9 - <26 g/day	≥26 g/day	Legumes	0 g/d	≥ 10 g/d
Nuts	<3 g/day	≥3 - <20 g/day	≥20 g/day	Nuts	0 g/d	≥ 15 g/d
Fish (not fried)	<13 g/day	13 g/day	>13 g/day	Fish$	0 g/d	≥ 15 g/d
Whole grains	<30 g/day	≥30 - <90 g/day	≥90 g/day	Whole grains	0 g/d and No consumption of whole grain products or ratio of whole grains to refined grains ≤0.7	≥ 90 g/d and No consumption of refined grain products or ratio of whole grains to refined grains ≥11
Poultry	<14 g/day	≥14 - <29 g/day	≥29 g/day			
Olive oil	<15 g/day	≥15 - <30 g/day	≥30 g/day	Fats and oils	No consumption of soft margarines, liquid cooking fats and vegetable oils or ratio of liquid cooking fats to solid cooking fats ≤0.6	No consumption of butter, hard margarines and cooking fats or ratio of liquid cooking fats to solid cooking fats ≥ 13
Butter and stick margarine	≥10 g/day	>5 - <10 g/day	≤5 g/day			
Full-fat cheese	≥30 g/day	>9 - <30 g/day	≤9 g/day	Dairy products*	0 g/d or ≥750 g/day	300−450 g/d
Red and processed meat	≥100 g/day	>43 - <100 g/day	≤43 g/day	Red meat	≥ 100 g/d	≤45 g/d
				Processed meat	≥ 50 g/d	0 g/d
Wine	>100 ml/d	NA	≤100 ml/d	Alcohol	Women: ≥ 20 g ethanol/d Men: ≥30 g ethanol/d	Women: ≤10 g ethanol/d Men: ≤10 g ethanol/d
				Tea	0 g/d	≥ 450 ml/d
				Coffee	Any consumption of unfiltered coffee	Consumption of only filtered coffee or no coffee consumption
				Sweetened beverages and fruit juices	≥250 g/day	0 g/d
				Salt	>3.8 Na/d	≤1.9 Na/d
Take out, fried foods and snacks	>3 serving eq/wk	>1 - ≤3 serving eq/wk	≤1 serving eq/wk	Unhealthy choices	>7 week choices/wk	≤ 3 week choices/wk
Cookies, pastries and sweets	>4 serving eq/wk	>2 - ≤4 serving eq/wk	≤2 serving eq/wk			

DHD2015-index: Dutch Healthy Diet 2015 index; MIND-NL: Dutch version of the Mediterranean-Dietary Approach to Stop Hypertension Intervention for Neurogenerative Delay Diet.

* maximum of 40 g cheese could be included.

$ maximum of 4 g lean fish could be included.

As a measure of adherence to the Dutch dietary guidelines, the DHD2015-index was calculated [16]. The DHD2015-index consists of 16 food components, representing the Dutch dietary guidelines, and an extra section comprising unhealthy choices. Food components are categorized in adequacy (healthy) food groups, ratio food groups (reflect replacement of less desired foods with healthier options), optimum food group, qualitative food group (focus on type of replacement instead of amount), and moderation (unhealthy) food groups. Adequacy (healthy) food groups are vegetables, fruit, whole grains, legumes, fish, nuts, and tea. Ratio food groups are fats and oils, and whole grains. Food groups with an optimum are dairy products. The qualitative component is coffee, which reflects type of coffee consumption. Moderation (unhealthy) food groups are red meat, processed meat, sweetened beverages and fruit juices, alcohol, salt, and unhealthy choices. Unhealthy choices include sweet spreads, cakes, cookies, chips or pretzels, chocolate, savoury snacks, sauces, and use of sugar in coffee or tea. Cut-off values for scoring are shown in Table 1. Each food group can be awarded a score between 0 and 10. For the DHD2015-index, a total score between 0 and 160 points can be given, with a higher score presenting better adherence to the Dutch food-based dietary guidelines.

Given that the short FFQ does not capture complete dietary intake and may insufficiently represent energy intake and certain nutrients, three-day food record data were used to obtain more detailed and comprehensive information on food groups and nutrient composition. Food records were administrated through a smartphone application (Traqq®) [17]. Three days were randomly assigned to the participant, i.e. two weekdays and one weekend day, at which participants received a notification at 08:00 AM each morning to track their food intake for the day using an extensive food list based on the Dutch Food Composition Database (NEVO 2016) [18]. Data of the food records were entered in the computation module of Compl-eat™ [19], where energy and nutrient intakes were calculated using the 2016 Dutch Food Composition Database. Food items were grouped into 47 food groups adapted from the GloboDiet food group categorization, taking into account the specific food groups of the MIND diet (e.g. berries).

### 2.3 Cognitive function

A global cognition composite score was derived from subtest scores of the neuropsychological test battery (NTB). The NTB included the 15-word verbal learning test delayed recall (episodic memory) [20], the Digit Symbol Substitution Test 90 seconds (processing speed) [21], the Wechsler Adult Intelligence Scale digit span backwards [22], and the semantic fluency test (animals; both attention and executive functions) [23,24]. A higher score of these tests indicates better performance. The raw scores of the individual tests were transformed to z-scores using the cohort-wide means and standard deviations (SD). The global cognition composite score was calculated by averaging the z-scores of the individual test scores and re-standardization. The minimum number of necessary individual tests was set to 3 out of 4 for calculating the total global cognition composite score.

Finally, the Montreal Cognitive Assessment (MoCA) was administrated for descriptive purposes rather than as outcome variable.

### 2.4 Covariates

Potential confounders were *a priori* selected based on previous literature [2,25]. Information on age, sex, level of education (low, middle, high; based on the International Standard Classification of Education (ISCED 2011) guidelines), smoking status (current, former, never), and resource-based socioeconomic status (SES; square root of average household income) was collected during the FINGER-NL baseline visit. Body weight and height were measured and BMI was calculated as weight (kg)/(height (m)^2^). Physical activity was assessed using the SQUASH questionnaire and categorised as meeting/not meeting Dutch physical activity guidelines of 2017 [26,27]. Cardiovascular risk factors (yes/no) were determined based on history of coronary heart disease (myocardial infarction, angina pectoris or vascular surgery concerning the heart), hypertension (use of antihypertensive medication or systolic blood pressure ≥140 or diastolic blood pressure ≥90 mmHg), hypercholesterolemia (LDL > 3.5 mmol/L or total cholesterol >5 mmol/L), and diabetes (medical history or HbA1c ≥ 6.5% / 48 mmol/mol).

### 2.5 Statistical analyses

Participant characteristics are presented as means and standard deviation (SD) for normally distributed variables, median and interquartile range (IQR) for non-normally distributed variables and percentages for categorial variables.

The associations between the dietary indices and cognition were tested by uni- and multi-variable linear regression analyses, for MIND-NL diet and DHD2015-index separately. Analyses were adjusted for age, sex, and education (model 1), and additionally for resource-based SES, BMI, physical activity level, smoking status, and cardiovascular risk factors (model 2). In each model, MIND-NL score or DHD2015-index were included in the model as continuous variable (estimating difference in cognition per 1-point increase in diet score). A two-sided p-value of ≤0.05 was considered statistically significant.

Previous literature has shown that physical activity level [28,29], sex [30,31], resource-bases SES [32], and age (below 70, and ≥70) [33] might moderate the association between MIND diet adherence and cognition. Therefore, the interaction terms between diet adherence and these variables were tested in the fully adjusted multiple regression model. Furthermore, we tested the interaction term between diet and education level. For the interaction analysis we dichotomized age (<70, and ≥70) based on previous literature [33]. Resource-based SES was dichotomized using the median as cut-off point (€2,569). A significant p-value for interaction was set at p ≤ 0.1.

Missing data of covariates (0.1–6.3 %) were imputed using multiple imputations with chained equations (with 10 imputation and 10 iterations, MICE package for R software [34]). The analyses were performed in each imputed dataset separately, and the estimates were subsequently pooled using Rubin’s rules [35]. Complete-case analyses are shown in Supplementary Tables S2 and S4.

### 2.6 Explorative analyses

To identify direct links between global cognitive function and individual dietary intake components, network analyses were performed via Copula Graphical Modelling (CGM). CGM offers a data-driven way to identify links in a dataset and has been optimised for nutritional research by allowing data to be non-normally distributed or highly correlated, such as is the case in nutritional epidemiology. The main benefit of using CGM are its unbiased way of correcting for every other variable in the dataset, that makes it an open-minded approach in finding links between nutrients and outcomes of interest in the dataset. The main limitation is that it only indicates the direction of found associations and no magnitude of effects, hampering conclusions on clinical relevance. The exact way in which CGM works, as well as R-packages and manuals can be retrieved elsewhere [36,37].

Two separate network analyses, with a nonparanormal transformation (method = "npn") via the nutriNetwork package (version 0.1.2), were performed to identify direct associations (i.e., associations not running via other dietary intake components) between dietary intake and global cognition score: one analysis was based on food groups and the other one on nutrients. Each network adjusted all associations for the confounders included in the fully adjusted linear regression models (model 2). Additionally, food group-global cognition relations were adjusted for all other food group intakes, and nutrient-global cognition relations for all other nutrient intakes. For each dataset (food groups, nutrients), we tested a range of penalty parameters (ρ) to generate candidate networks. The optimal sparse network was then selected with the selectnet() function, which uses the extended Bayesian information criterion (EBIC). Certainty was quantified as the average edge certainty of 100 bootstrap iterations per dataset.

All analyses were conducted using R version 4.3.1.

## 3 Results

### 3.1 Participant characteristics

Of the 1,210 participants, a total of 74 participants were excluded since dietary data of the FQQ were missing and one person was excluded due to incomplete neuropsychological tests. Therefore, the current analyses comprised 1,135 participants.

Participant characteristics are shown in Table 2. The median age of participants was 67 [IQR: 64−71] years, the median MoCA score was 27 [IQR: 25−28], and 63.3% were female. On average, participants had a BMI of 28.2 (SD 4.2) kg/m^2^. Participants’ mean score of the MIND-NL diet and DHD2015-index was 8.4 (SD 1.8, range 2.0–14.0) and 106.3 (SD 17.4, range 52.0–150.0), respectively. On average, participants with lowest adherence to the diet scores were less educated, met the physical activity guidelines less often, and were more often smokers.

**Table 2:** Participant characteristics.

Characteristic	N total	Mean^1^	SD^1^
MIND-NL score	1,135	8.4	1.8
DHD2015-index	1,135	106.3	17.4
Age (years), median [IQR]	1,135	67	64−71
Females, n (%)	1,135	718	63.3
Resource-based SES (€)	1,064	2699	959
Marital status, cohabiting, n (%)	1,135	779	68.6
Level of education, n (%)	1,134		
Low		155	13.7
Middle		282	24.9
High		697	61.4
BMI (kg/m^2^)	1,134	28.2	4.2
Energy intake (kcal)	1,077	1644	482
Adherent to physical activity guidelines*, n (%)	1,121	129	11.5
Smoking status n(%)	1,135		
Current		48	4.2
Former		711	62.6
Never		376	33.1
Hypertension#, n (%)	1,135	786	69.3
Hypercholesterolemia$, n (%)	1,131	692	61.2
Coronary heart disease^, n (%),	1,135	149	13.1
Diabetes&, n (%)	1,135	116	10.2
MoCA score (/30), median [IQR]	1,134	27	25−28
15-word verbal learning test, delayed recall (n words), median [IQR]	1,135	7.7	3.5
Digit Symbol Substitution Test (n symbols in 90 s)	1,131	50.5	9.9
Digit span backward test (/14)	1,135	6.6	1.9
Animal fluency (n animals)	1,135	25.2	5.6

1 Values are presented as mean ± SD, unless indicated otherwise. DHD2015-index: Dutch Healthy Diet 2015 index; MIND-NL: Dutch version of the Mediterranean-Dietary Approach to Stop Hypertension Intervention for Neurogenerative Delay Diet; MOCA: Montreal Cognitive Assessment; SES: Socioeconomic Status; 15WT: 15 word test.

* Physical activity guideline: At least 150 min moderate to vigorous aerobic activity and at least two days of bone- and muscle strengthening activities.

# Hypertension: systolic BP ≥ 140 mmHg and/or diastolic BP ≥ 90 mmHg and/or use of antihypertensive drugs.

$ Hypercholesterolemia defined as LDL > 3.5 mmol/L and/or total cholesterol >5 mmol/L.

^ Coronary heart disease includes history of myocardial infarction, angina pectoris, and/or vascular surgery concerning the heart.

& Diabetes defined as history of diabetes and/or HbA1c is 6.5% (48 mmol/mol) or higher.

### 3.2 Multi-variable linear regression analyses

**Fig. 1:**
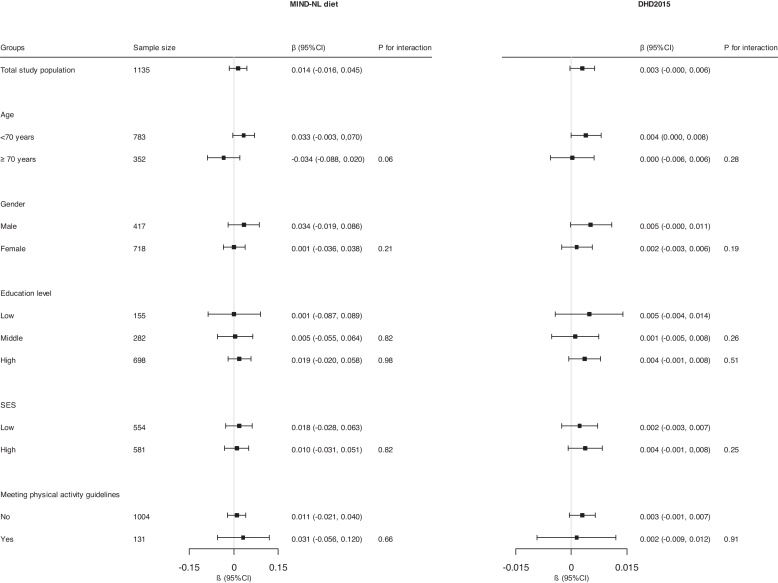
Association between MIND-NL diet and DHD2015-index with global cognition score, according to subgroups of age, sex, education level, SES, and meeting physical activity guidelines. Betas are depicted as 1 point increase in the dietary score. The analyses are adjusted for age, sex (except when stratified), education level (except when stratified), Socioeconomic status (SES), BMI (in kg/m2), energy intake (kcal), physical activity level (met/not met, except when stratified), smoking status (never, current, former), and cardiovascular risk factors (yes/no; hypertension, hypercholesterolemia, coronary heart disease, diabetes).

After adjustment for confounders, neither the MIND-NL diet nor the DHD2015-index was significantly associated with the global cognition composite score (β = 0.014, 95%CI: -0.016, 0.045, p = 0.35 and β = 0.003, 95%CI: -0.000, 0.006, p = 0.07, respectively) (Table 3, model two, Fig. 1).

**Table 3:** Cross-sectional associations of MIND-NL diet and DHD2015-index with global cognition, with use of multiple imputations (n = 1,135).

Dietary pattern	Crude model			Model 1			Model 2		
	beta	95% CI	p-value	beta	95% CI	p-value	beta	95% CI	p-value
MIND-NL	**0.050**	**0.018, 0.081**	**0.005**	0.013	−0.016, 0.043	0.37	0.014	−0.016, 0.045	0.35
DHD2015	**0.008**	**0.005, 0.011**	**<0.001**	0.003	−0.000, 0.006	0.06	0.003	−0.000, 0.006	0.07

DHD2015-index: Dutch Healthy Diet 2015 index; MIND-NL: Dutch version of the Mediterranean-Dietary Approach to Stop Hypertension Intervention for Neurogenerative Delay Diet. Model 1: adjusted for age, sex, education level Model 2: adjusted for model 1 + SES, BMI (in kg/m^2^), energy intake (kcal), physical activity level (met/not met), smoking status (never, current, former), and cardiovascular risk factors (yes/no; hypertension, hypercholesterolemia, coronary heart disease, diabetes) Beta’s are depicted as 1 point increase in the dietary score.

Interaction analyses showed that the association between MIND-NL diet score and global cognition was not consistent for different age groups (p_interaction_ = 0.06). Among those aged <70 years, a positive trend was observed in the association between MIND-NL diet and global cognitive score (β = 0.033, 95%CI: -0.003, 0.070, p = 0.08). Among participants aged <70 years, the association between DHD2015-index and global cognition was significant (β = 0.004, 95%CI: 0.000, 0.008, p = 0.048), although there was no significant interaction with age (p_interaction_ = 0.28) (Fig. 1). For other subgroup analyses, no interactions were found.

With respect to the individual cognitive tests, there was an association between DHD2015-index and animal fluency (β = 0.021, 95%CI 0.001, 0.040, p = 0.04). No other associations were found for MIND-NL and DHD2015-index with individual cognitive tests (Supplementary Table S3).

### 3.3 Network analyses

Table 4 shows the direct correlations between food groups and nutrients with global cognition. The group *fruiting vegetables* was positively correlated with global cognition with a certainty of 99% based on bootstrap analysis. This association was non-linear, positive associations were observed from 100 grams daily onwards and optimal intake was seen between 200 and 400 grams/day (Supplementary Figure S4). Of the nutrients, vitamin E was positively correlated with global cognition with a 100% certainty. The optimal intake of vitamin E estimated between 15 and 20 mg/day (Supplementary Figure S5). For both network analyses, being female and highly educated were positively correlated with global cognition, whereas age was negatively correlated, with all having 100% certainty on bootstrap analysis.

**Table 4:** Food groups and Nutrients with direct relations to global cognition, adjusted for all variables in dataset.

	Relation to Cognition	%Certainty		Relation to Cognition	%Certainty
Food groups			Nutrients		
**Sex**		**100**	**Sex**		**100**
**Age**		**100**	**Age**		**100**
**Education**		**100**	**Education**		**100**
**FruitingVeg**		**99**	**Vitamin E**		**100**
*HighFatCheese*		*87*	*SES*		*85*
*Sauces*		*84*	*EPA*		*82*
*SES*		*84*	*Water*		*74*
*LeekOnionGarlic*		*83*	*HaemIron*		*65*
*Berries*		*68*	*BMI*		*44*
*Juices*		*57*			
*LiquidFats*		*52*			
*Seeds*		*51*			
*Butter*		*45*			
*Nuts*		*44*			
*Water*		*6*			

Certainty levels ≥ 95% are depicted in bold. Associations are corrected for all other variables in the dataset: Dataset with food groups: n = 47 food groups, n = 9 confounders; Dataset with nutrients: n = 47 nutrients, n = 9 confounders. Categorisation variables: sex (1 = male, 2 = female), education level (categorised as low, medium, or high), adherence to physical activity guidelines (0 = no, 1 = yes), smoking status (1 = daily, 2 = non-daily, 3 = former, 4 = never, with higher values indicating lower exposure), cardiovascular risk (0 = no risk, 1 = presence of hypertension, hypercholesterolemia, coronary heart disease, or diabetes).

## 4 Discussion

In the current cross-sectional study among Dutch older adults at risk of cognitive decline, no association was found between adherence to either the MIND-NL diet or the Dutch dietary guidelines (DHD2015-index) and global cognitive function. However, age moderated the association between MIND-NL diet adherence and global cognitive function, with older adults under 70 years of age showing a positive trend. Among older adults under 70 years of age, higher adherence to the Dutch dietary guidelines was significantly associated with better global cognitive function.

Previous studies have predominantly reported positive cross-sectional associations between the MIND diet and global cognition composite scores [25,[38], [39], [40], [41], [42]]. The discrepancy between our findings and those of previous studies might be due to differences in study populations. Our study is the first to include a population at risk of cognitive decline, while the other studies included a general population of older adults. Therefore, the effect of other risk factors in our population may have outweighed the effect of diet. Furthermore, our study is the first to examine this association in a European population, specifically a Dutch population. Since the MIND diet is a food-based dietary pattern, it is influenced by cultural differences in eating habits. Previous studies have primarily been conducted in U.S. populations. Since the original food groups were kept within the MIND-NL diet score, cultural differences in eating habits compared to the U.S. population may have weakened the strength of the association, potentially explaining our null finding. One Chinese study found a positive association between MIND diet and global cognition. In contrast to the MIND-NL diet, the Chinese MIND diet has been significantly adapted to local dietary habits, making the diet more culturally appropriate and thereby potentially strengthening its association with cognitive function [42]. For the future, further adaptations to the MIND-NL could be considered, such as adding other types of vegetables next to green leafy vegetables, possibly deleting the berry component (low consumption in the Netherlands), or adding beverages.

Although the association between DHD2015-index and global cognition was insignificant, we found a significant positive association with the cognitive domain attention and executive function, as measured by the animal fluency test. Furthermore, in adults under 70 years, a significant positive association between DHD2015-index and global cognition was observed. The DHD2015-index was specifically developed to measure adherence to the Dutch dietary guidelines, which are based on the dietary intake pattern of the Dutch population and the required food products to achieve health gain in the Netherlands [43]. Therefore, its food group composition may better align with the eating habits of our study population. Furthermore, while the Dutch dietary guidelines did not take evidence regarding neurodegenerative diseases into account, they aim to tackle risk factors of dementia including hypertension, high LDL cholesterol, and diabetes [1]. Especially, these risk factors are relevant in our study population, as the FINGER-NL study used the modifiable risk factors hypertension and high cholesterol as one of the inclusion criteria [11].

Our sub-analyses suggest that age might be an important moderator in the association between diet and global cognition. A previous study found that among older participants aged ≥70 years, a higher MIND diet score was associated with reduced risk of subjective memory complaints over a mean follow-up period of six-years [33]. De Crom et al. observed that the association of MIND diet was slightly larger in Dutch participants aged <75 years compared to those aged ≥75 years [7]. Our results are in line with this latter study, as we mainly observed positive trends in individuals under 70 years of age. Our results might be explained by the change in physiological needs and metabolism with age. With ageing, our digestive system becomes less efficient in absorbing certain nutrients e.g. vitamin B12, as a result of slower return of postprandial-raised gastric pH to acidic pH and bacterial overgrowth [44,45]. Additionally, the intake of medication, which is highly prevalent among older adults, affects absorption of nutrients or increases nutrient losses [45]. Furthermore, with ageing, low-grade (neuro)inflammation arises, which might ask for increased intake of antioxidants. Due to these physiological changes, it might be that both adherence to the MIND-NL diet and Dutch dietary guidelines do not deliver sufficient nutrients to the more aged (≥70 years) population to preserve cognitive function. However, this age-specific effect, might also be a result of a more selective group of cardiovascular disease survivors among those aged ≥70 years.

In the explorative network analyses, using Copula Graphical Models, we identified dietary intake of the food group *fruiting vegetables* to be directly correlated with global cognitive function. Fruiting vegetables (e.g. tomatoes, red bell peppers, and eggplant) are rich in different types of carotenoids with antioxidant function. Fruiting vegetables might also represent the total vegetable group in general, as fruiting vegetables were the vegetable group with highest intake and variation in our population. Another interesting finding in the network analyses is that high fat cheese showed a positive trend with global cognition (certainty: 87%), while the MIND diet scored inversely on this component. Contrary to the MIND diet, the DHD2015-index did not penalize cheese intake and took a maximum of 40 grams/day of cheese into account in the dairy component. According to its development, high fat cheese was included in the MIND diet due to its contribution in intake of saturated fatty acids. Based on our results, it seems that saturated fatty acids are not one of the main driving factors in relation to a lower global cognitive composite score in our study population. A possible non-linear association between high-fat cheese and cognitive functioning [46] may also explain the contradictory findings.

Based on the network analysis using the nutrient database, vitamin E was positively correlated with global cognition. The observed association for vitamin E aligns with existing literature on cognitive decline and dementia [47]. Our observed optimum intake of 15−20 mg vitamin E per day is in line with results of the Dutch Rotterdam study [48]. Vegetables are a source of vitamin E, but nuts, seeds, and vegetable oils are richer sources of vitamin E. Of the vegetable groups, green leafy, fruiting, and cruciferous vegetables are most rich in vitamin E. Although the network analyses give valuable insights, sensitivity analyses did not fully confirm the results and the results should still be confirmed in another Dutch cohort (Supplementary Table S5). Nonetheless, these findings can serve as a basis for further investigation into the most effective dietary patterns for cognitive function among older adults in the Netherlands.

One of the limitations of the current study is its cross-sectional design. Although it allows for trend identification, it excludes the temporal study of cognitive changes over time and drawing of causal inferences. A future study, using data of the FINGER-NL study, can be used to explore the importance and consequently weighting of MIND components in relation to cognitive decline. Another limitation is that our study predominantly involved participants with middle to higher levels of education. Since individuals with lower educational attainment tend to have poorer dietary habits, our results cannot be generalized to the broader population. Another limitation is that the apolipoprotein (APOE) genotype has not been taken into account in the current analyses, as data were not available during the time of analysis. Although the role of *APOE4* is not yet fully understood, it is a well-known risk factor for dementia involved in the cholesterol pathway. Therefore, *APOE4* can moderate the association between diet and cognition [49]. A final limitation that should be acknowledged is that a 72-item FFQ may have limited sensitivity in capturing full dietary diversity, especially for micronutrients.

The strengths of this study are the detailed dietary data, group size, known risk profile, comprehensive cognitive assessment, and the explorative analysis to better understand the importance of individual food groups in relation to global cognition. Although we included an at-risk population of cognitive decline, the average score and standard deviation of the DHD2015-index were comparable to those reported in other studies including a general Dutch population [14,50]. This suggests that the risk profile had not altered dietary habits in our study population.

To conclude, this study observed that adherence to the Dutch dietary guidelines was associated with better global cognition among older adults under the age of 70 years at risk of cognitive decline. Age moderated the relationship between the MIND-NL diet and global cognition, with a positive trend observed in adults younger than 70. This study did not find added value of the MIND-NL diet, a tailored dietary pattern specifically developed for delay of neurodegenerative diseases, compared to the Dutch dietary guidelines. Findings are hypothesis-generating and based on cross-sectional observations, pending longitudinal confirmation from the longitudinal FINGER-NL study. Future research might investigate whether the MIND-NL diet food groups should be further modified to better resemble the Dutch food culture, which could in turn increase the neuroprotective potential of the dietary pattern in the Netherlands. Furthermore, future research should further explore the moderating effect of age.

## Credit authorship contribution statement

The authors’ contributions were as follows: SB, MS, YV, and LG were involved in conceptualisation of the study. SB conducted the initial analyses and PG performed the explorative analyses. SB, MS, YV, and LG contributed to the interpretation of the data. All authors were involved in the design and data-collection of FINGER-NL and critically reviewed this paper’s content. All have approved the final version submitted for publication.

## Financial support

FINGER-NL is part of MOCIA, which received a Crossover grant (MOCIA 17611) from the Dutch Research Council (NWO). The MOCIA program is a public–private partnership (see https://mocia.nl/scientific/).

## Declaration of competing interest

No conflicts of interest were reported by the authors.
