# Supplementary material for: The association between the MIND-NL diet, Dutch dietary guidelines, and global cognitive function in an older population at risk for cognitive decline
Source: J Nutr Health Aging. 2025 Sep 8;29(11):100680. doi: 10.1016/j.jnha.2025.100680 (PMC12451163; doi:10.1016/j.jnha.2025.100680)
Supplement: Supplementary file 1 [file mmc1.pdf]

The association between the MIND-NL diet, Dutch dietary guidelines, and global cognitive function in an older population at risk for cognitive decline.
Sonja Beers

Supplementary Table 1. Baseline characteristics according diet tertiles

Characteristic	Overall (n=1135) Mean[1]	SD[1]	MIND-NL diet score tertiles[*] T1 (n=441) Mean[1]	SD[1]	T2 (n=389) Mean[1]	SD[1]	T3 (n=305) Mean[1]	SD[1]	p-value	DHD2015 index tertiles[*] T1 (n=396) Mean[1]	SD[1]	T2 (n=376) Mean[1]	SD[1]	T3 (n=363) Mean[1]	SD[1]	p-value
MIND-NL score	8.4	1.8	6.7	1.0	8.7	0.6	10.7	0.9	**<0.001**	7.2	1.5	8.5	1.4	9.8	1.5	**<0.001**
DHD2015-index	106.3	17.4	95.6	15.2	107.8	13.8	119.7	14.2	**<0.001**	88.2	10.4	107.3	6.6	124.8	8.9	**<0.001**
Age (years), median [IQR]	67	64-71	67	64 - 70	67	64 - 71	67	64 - 70	0.29	66	64 - 70	67	64 - 71	67	64 - 71	**0.02**
Females, n(%)	718	63.3	300	68.0	221	56.8	197	64.4	**0.003**	252	63.6	241	64.1	225	62.0	0.82
SES (n=1064)	2699	959	2645	961	2673	949	2811	962	0.06	2700	956	2663	968	2735.8	954	0.61
Material status, cohabiting, n(%)	779	68.6	300	68.0	276	0.71	203	66.6	0.43	277	69.9	255	67.8	247	68.0	0.88
Level of education, n(%)									**<0.001**							**<0.001**
Low																
Middle	155	13.7	77	17.5	51	13.1	27	8.9		64	16.2	46	12.2	45	12.4	
High	282	24.9	129	29.3	93	23.9	60	19.7		125	31.6	86	22.9	71	19.6	
(n=1134)	697	61.4	235	53.3	245	63.0	217	71.4		207	52.3	244	64.9	246	68.0	
BMI (kg/m^2) (n=1134)	28.2	4.2	28.4	4.4	28.0	4.0	28.2	4.2	0.52	28.6	4.4	28.2	4.1	27.8	4.1	**0.03**
Energy intake, kcal (n=1077)	1644	482	1658	493	1654	462	1612	488	0.41	1656	499	1606	459	1672	484	0.17
Adherent to physical activity guidelines, n(%) (n=1121)	129	11.5	33	7.6	48	12.6	48	15.9	**0.002**	32	8.1	44	12.0	53	14.7	**0.02**
Smoking status, n(%)									**<0.001**							**<0.001**
Current	48	4.2	31	7.0	15	3.9	2	0.7		29	7.3	13	3.5	6	1.7	
Former	711	62.6	289	65.5	242	62.2	180	59.0		250	63.1	248	66.0	213	58.7	
Never	376	33.1	121	27.4	132	33.9	123	40.3		117	29.5	115	30.6	144	39.7	
Hypertension[#], n(%)	786	69.3	305	69.2	266	68.4	215	70.5	0.84	273	68.9	275	73.1	238	65.6	0.08
Hypercholesterolemia[$], n(%) n=1131	692	61.2	253	57.8	246	63.4	193	63.3	0.17	232	59.0	235	62.7	225	62.0	0.55
Coronary heart disease[^], n (%)	149	13.1	60	13.6	47	12.1	42	13.8	0.75	48	12.1	60	16.0	40	11.0	0.13
Diabetes[&], n(%)	116	10.2	51	11.6	32	8.2	33	10.8	0.26	45	11.4	35	9.3	36	9.9	0.62

MoCA score, median [IQR] (n=1134)	27	25-28	27	25 - 28	27	25 - 28	27	25 - 28	0.26	27	25 - 28	27	25 - 28	27	26 - 28	0.14
15-word verbal learning test, delayed recall (n words)	7.7	3.5	7.8	3.6	7.8	3.3	7.6	3.5	0.75	7.6	3.6	7.8	3.5	7.8	3.3	0.47
Digit Symbol Substitution Test (n symbols) (n=1131)	50.5	9.9	50.1	10.0	50.3	10.0	51.5	9.6	0.13	50.3	10.0	50.5	9.5	50.8	10.2	0.75
Digit span backward test	6.6	1.9	6.5	1.8	6.7	1.9	6.5	1.8	0.43	6.5	1.8	6.6	2.0	6.6	1.8	0.66
Animal fluency (n animals)	25.2	5.6	24.9	5.6	25.2	5.6	25.8	5.6	0.07	24.9	5.5	25.2	5.8	25.7	5.5	0.09

* Tertiles are sex-specific tertiles, range of tertiles: MIND T1: 2.0-8.0, T2: 7.5-9.5, T3: 9.5-14.0. DHD15 T1: 52-103 , T2: 93-118 , T3: 150

Hypertension: systolic BP >=140 and/or diastolic BP >=90 mmHg and/or use of antihypertensive drugs

$ Hypercholesterolemia defined as LDL >3.5 and/or total cholesterol >5

^ Coronary heart disease include history of myocardial infarction, angina pectoris, and/or vascular surgery concerning the heart

& Diabetes defined as history of diabetes and/or HbA1c is 6.5% (48 mmol/mol) or higher

Supplementary Table 2. Cross-sectional associations of MIND-NL diet and DHD2015-index with global cognition, complete-cases

Dietary pattern	Crude model n=1,135			Model 1 n=1,134			Model 2 n=1,048		
	beta	95% CI	p-value	Beta	95% CI	p-value	beta	95% CI	p-value
**MIND-NL**	**0.050**	**0.018, 0.081**	**0.002**	0.014	-0.016, 0.044	0.35	0.012	-0.019, 0.044	0.44
**DHD2015**	**0.008**	**0.005, 0.011**	**<0.001**	**0.003**	**0.000, 0.007**	**0.05**	0.003	-0.000, 0.006	0.09

DHD2015-index: Dutch Healthy Diet 2015 index; MIND-NL: Dutch version of the Mediterranean-Dietary Approach to Stop Hypertension for Neurogenerative Delay Diet.

Model 1: adjusted for age, sex, education level

Model 2: adjusted for model 1 + SES, BMI (in kg/m2), physical activity level (met/not met), smoking status (never, current, former), and cardiovascular risk factors (yes/no; hypertension, hypercholesterolemia, coronary heart disease, diabetes)

Beta's are depicted as 1 point increase in the dietary score.

Supplementary Table 3. Cross-sectional associations of MIND-NL diet and DHD2015-index with individual cognitive tests, with use of multiple imputations

Dietary pattern	Crude model			Model 1			Model 2		
	beta	95% CI	p-value	beta	95% CI	p-value	beta	95% CI	p-value
**MIND-NL**									
15WTdr	**0.16**	**0.047, 0.27**	**0.005**	0.036	-0.068, 0.14	0.50	0.037	-0.069, 0.14	0.49
DSST90	**0.43**	**0.12, 0.75**	**0.007**	0.19	-0.11, 0.50	0.21	0.13	-0.18, 0.44	0.41
Animal fluency	**0.24**	**0.06, 0.42**	**0.008**	0.11	-0.064, 0.29	0.21	0.13	-0.049, 0.31	0.15
WAISdsB	-0.008	-0.067, 0.051	0.79	-0.030	-0.090, 0.030	0.32	-0.020	-0.081, 0.040	0.51
**DHD2015**									
15WTdr	**0.029**	**0.018, 0.041**	**<0.001**	0.011	-0.001, 0.022	0.062	0.010	-0.0015, 0.022	0.09
DSST90	**0.037**	**0.0038, 0.071**	**0.02**	0.0084	-0.025, 0.042	0.63	-0.0027	-0.037, 0.031	0.88
Animal fluency	**0.035**	**0.016, 0.053**	**<0.001**	0.019	-0.000, 0.038	0.053	**0.021**	**0.001, 0.040**	**0.04**
WAISdsB	0.0032	-0.003, 0.009	0.32	0.0010	-0.006, 0.008	0.75	0.0025	-0.004, 0.009	0.46

DHD2015-index: Dutch Healthy Diet 2015 index; DSST90: Digit Symbol Substitution Test; MIND-NL: Dutch version of the Mediterranean-Dietary Approach to Stop Hypertension for Neurogenerative Delay Diet; WAISdsB: Wechsler Adult Intelligence Scale digit span backwards; 15WTdr: 15-word verbal learning test, delayed recall.

Model 1: adjusted for age, sex, education level

Model 2: adjusted for model 1 + SES, BMI (in kg/m^2), energy intake (kcal), physical activity level (met/not met), smoking status (never, current, former), and cardiovascular risk factors (yes/no; hypertension, hypercholesterolemia, coronary heart disease, diabetes)

Supplementary Table 4. Cross-sectional associations of MIND-NL diet and DHD2015-index with individual cognitive tests, complete cases

**Dietary pattern**	**Crude model**				**Model 1**				**Model 2**			
	N	beta	95% CI	p-value	N	beta	95% CI	p-value	N	beta	95% CI	p-value
**MIND-NL**												
15WTdr	1135	**0.16**	**0.047, 0.27**	**0.005**	1134	0.040	-0.064, 0.14	0.45	1048	0.033	-0.078, 0.144	0.56
DSST90	1131	**0.44**	**0.12, 0.75**	**0.007**	1130	0.20	-0.11, 0.51	0.21	1044	0.081	-0.239, 0.402	0.62
Animal fluency	1135	**0.24**	**0.063, 0.42**	**0.008**	1134	0.12	-0.061, 0.30	0.20	1048	0.131	-0.053, 0.315	0.16
WAISdsB	1135	-0.008	-0.067, 0.051	0.80	1134	-0.030	-0.090, 0.030	0.33	1048	-0.020	-0.083, 0.043	0.53
**DHD2015**												
15WTdr	1135	**0.029**	**0.018, 0.041**	**<0.001**	1134	0.011	-0.000, 0.023	0.05	1048	0.010	-0.002, 0.022	0.11
DSST90	1131	**0.038**	**0.0043, 0.071**	**0.03**	1130	0.0098	-0.024, 0.043	0.56	1044	-0.004	-0.039, 0.031	0.84
Animal fluency	1135	**0.035**	**0.016, 0.053**	**<0.001**	1134	**0.020**	**0.001, 0.039**	**0.04**	1048	0.018	-0.002, 0.038	0.08
WAISdsB	1135	0.0032	-0.003, 0.009	0.32	1134	0.0011	-0.0054, 0.0076	0.74	1048	0.002	-0.005, 0.009	0.61

DHD2015-index: Dutch Healthy Diet 2015 index; DSST90: Digit Symbol Substitution Test; MIND-NL: Dutch version of the Mediterranean-Dietary Approach to Stop Hypertension for Neurogenerative Delay Diet; WAISdsB: Wechsler Adult Intelligence Scale digit span backwards; 15WTdr: 15-word verbal learning test, delayed recall.

Model 1: adjusted for age, sex, education level.

Model 2: adjusted for model 1 + SES, BMI (in kg/m^2), physical activity level (met/not met), smoking status (never, current, former), and cardiovascular risk factors (yes/no; hypertension, hypercholesterolemia, coronary heart disease, diabetes)

Supplementary Table 5. Food groups and nutrients with direct relations to cognition, adjusted for all variables in dataset, including sensitivity analysis for sex, age and socioeconomic status (SES)

	Relation to Cognition	%Certainty	Men	Women	<70 years	≥70 years	Low SES	High SES
Food groups								
**Sex**	▲	**100**			**+100**	**+99**	**+100**	**+100**
**Age**	▼	**100**	**-100**	**-100**	**-100**	-8	**-100**	**-100**
**Education**	▲	**100**	**+99**	**+100**	**+100**	**+100**	**+98**	**+100**
**FruitingVeg**	▲	**99**	+9	+74	+81	+87	**+98**	+66
HighFatCheese	▲	*87*	*+87*	*+63*	*+56*	***+98***	***+99***	*+36*
Sauces	▲	*84*		*+92*	*+93*		*+89*	*+72*
SES	▲	*84*	*+85*	*+75*		***+99***		
LeekOnionGarlic	▲	*83*	*+69*	*+66*	*+88*		*+86*	
Berries	▲	*68*	*+79*		*+81*		*+52*	
Juices	▲	*57*		*+75*	*+62*			*+74*
LiquidFats	▲	*52*	***+95***			*+84*		***+96***
Seeds	▲	*51*	*+68*			*+55*		*+78*
Butter	▲	*45*		*+48*	*+62*		*+91*	
Nuts	▲	*44*			*+43*			
Water	▲	*6*		*+62*		***+97***		*+82*
GreenLeafyVeg			*+81*					*+74*
Olives			***+96***	*-64*		*+64*		
SugarSweets						*+81*		
DairySubs					*+42*			*+86*
Tubers			*-72*			*-63*		*-89*
Nutrients								
**Gender**	▲	**100**			**+100**	+9	**+100**	**+100**
**Age**	▼	**100**	**-96**	**-100**	**-100**		**-100**	**-98**
**Education**	▲	**100**	**+97**	**+100**	**+100**	+71	+89	**+95**
**Vitamin E**	▲	**100**	+93	+66	+76	+62	+59	**+99**
SES	▲	*85*	*+51*	*+61*		*+88*		
EPA	▲	*82*		*+53*	*+86*			
Water	▲	*74*		*+67*				*+47*

HaemIron	▼	*65*	*-69*	
BMI	▼	*44*		*-37*

Certainty levels ≥ 95% are depicted in bold. Associations are corrected for all other variables in the dataset: Dataset with food groups: n=47 food groups, n=9 confounders; Dataset with nutrients: n=47 nutrients, n=9 confounders.

Categorisation variables: sex (1 = male, 2 = female), education level (categorised as low, medium, or high).

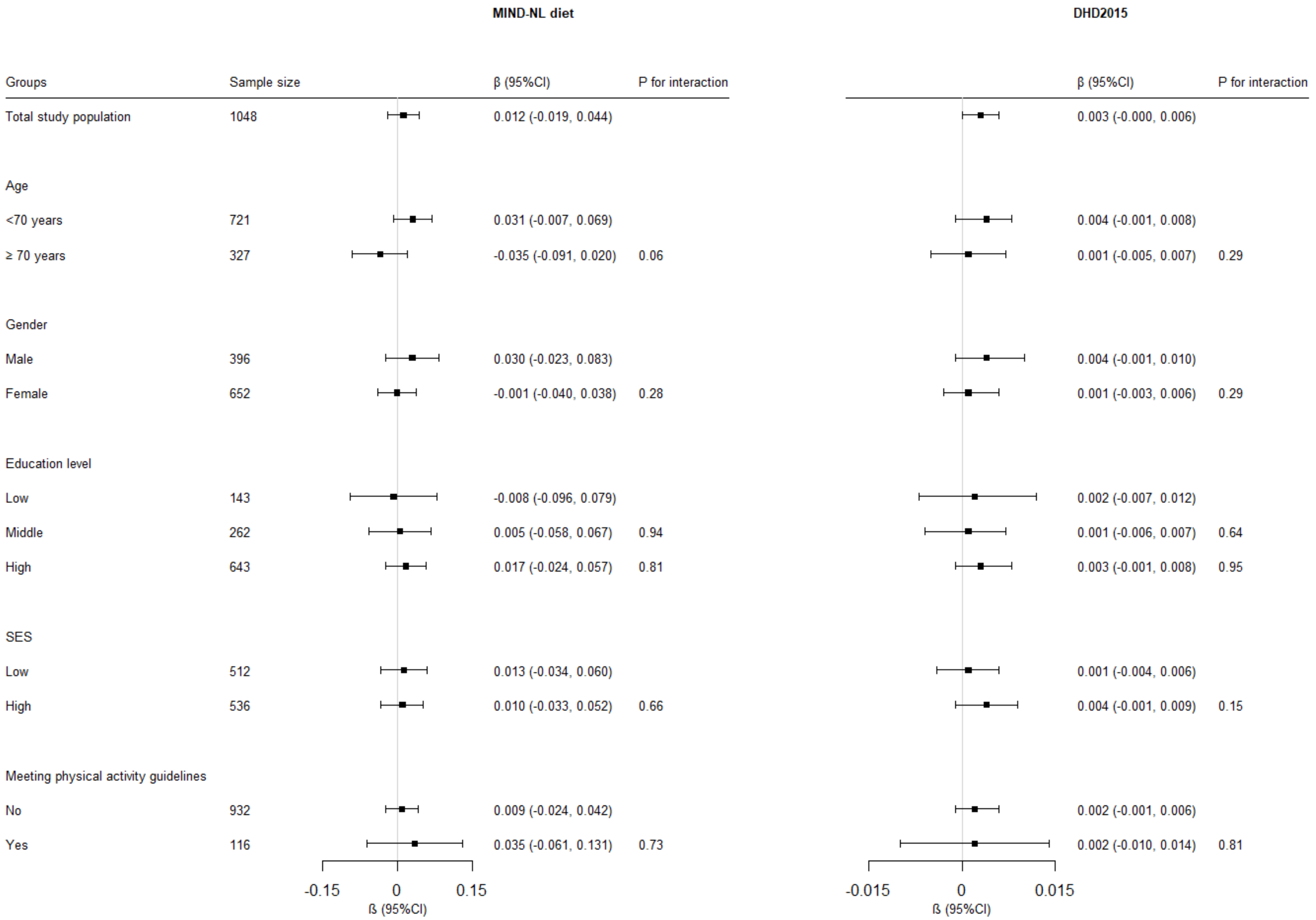

Supplementary Figure 1. Association between MIND-NL diet and DHD2015 index with global cognition score, according to subgroups of age, gender, education level, SES, and meeting physical activity guidelines (**complete cases**). Beta's are depicted as 1 point increase in the dietary score.
The analyses are adjusted for age, sex (except when stratified), education level (except when stratified), Socioeconomic Status (SES), BMI (in kg/m2), physical activity level (met/not met, except when stratified), smoking status (never, current, former), and cardiovascular risk factors (yes/no; hypertension, hypercholesterolemia, coronary heart disease, diabetes).

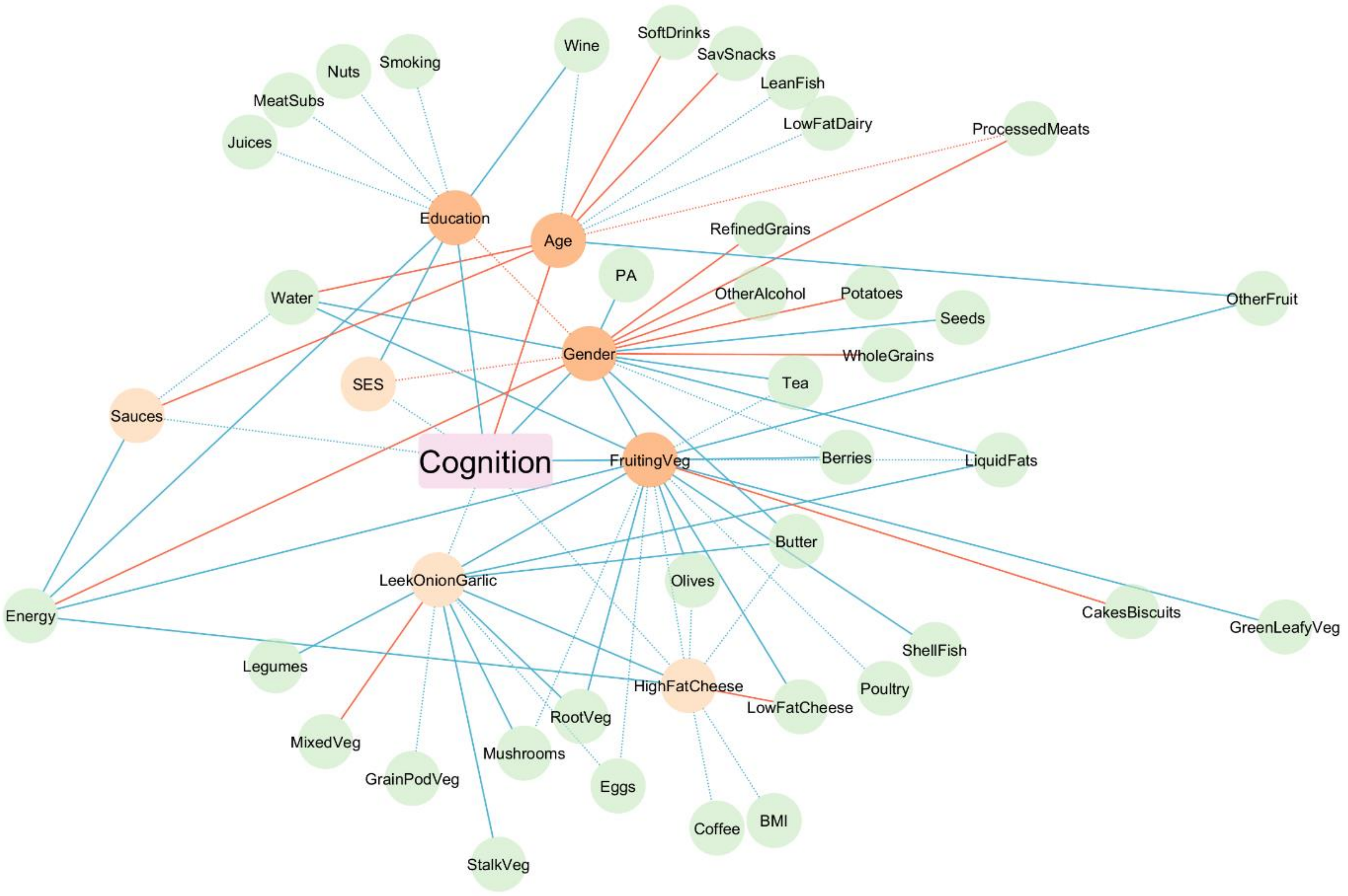

Supplementary figure 2. Network of food groups and their associations with cognition. This visualisation includes only first- and second-order links to cognition that exceed an 80% certainty threshold. Positive associations are shown in blue, negative associations in red. Dotted lines represent associations with 80–95% certainty, while solid lines indicate 95% or higher certainty.

Categorisation variables: sex (1 = male, 2 = female), education level (categorised as low, medium, or high), adherence to physical activity guidelines (0 = no, 1

= yes), smoking status (1 = daily, 2 = non-daily, 3 = former, 4 = never, with higher values indicating lower exposure), cardiovascular risk (0 = no risk, 1 = presence of hypertension, hypercholesterolemia, coronary heart disease, or diabetes).

This network visualisation was made using the final adjacency matrix (reflecting average edge certainty) which was exported to Cytoscape (1).

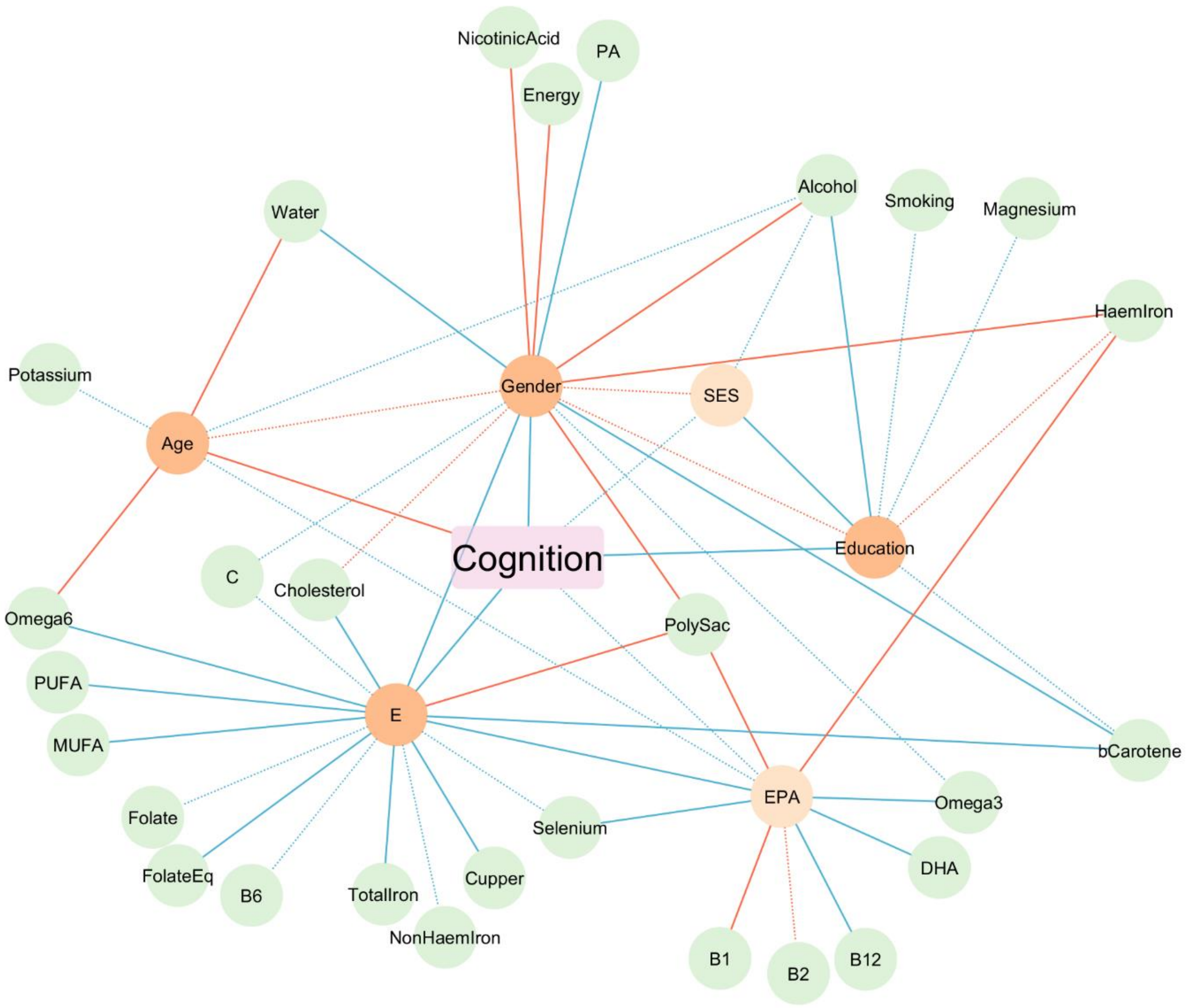

Supplementary figure 3. Network of nutrients and their associations with cognition. This visualisation includes only first- and second-order links to cognition that exceed an 80% certainty threshold. Positive associations are shown in blue, negative associations in red. Dotted lines represent associations with 80–95% certainty, while solid lines indicate 95% or higher certainty.

Categorisation variables: sex (1 = male, 2 = female), education level (categorised as low, medium, or high), adherence to physical activity guidelines (0 = no, 1 = yes), smoking status (1 = daily, 2 = non-daily, 3 = former, 4 = never, with higher values indicating lower exposure), cardiovascular risk (0 = no risk, 1 =

presence of hypertension, hypercholesterolemia, coronary heart disease, or diabetes).
This network visualisation was made using the final adjacency matrix (reflecting average edge certainty) which was exported to Cytoscape (1).

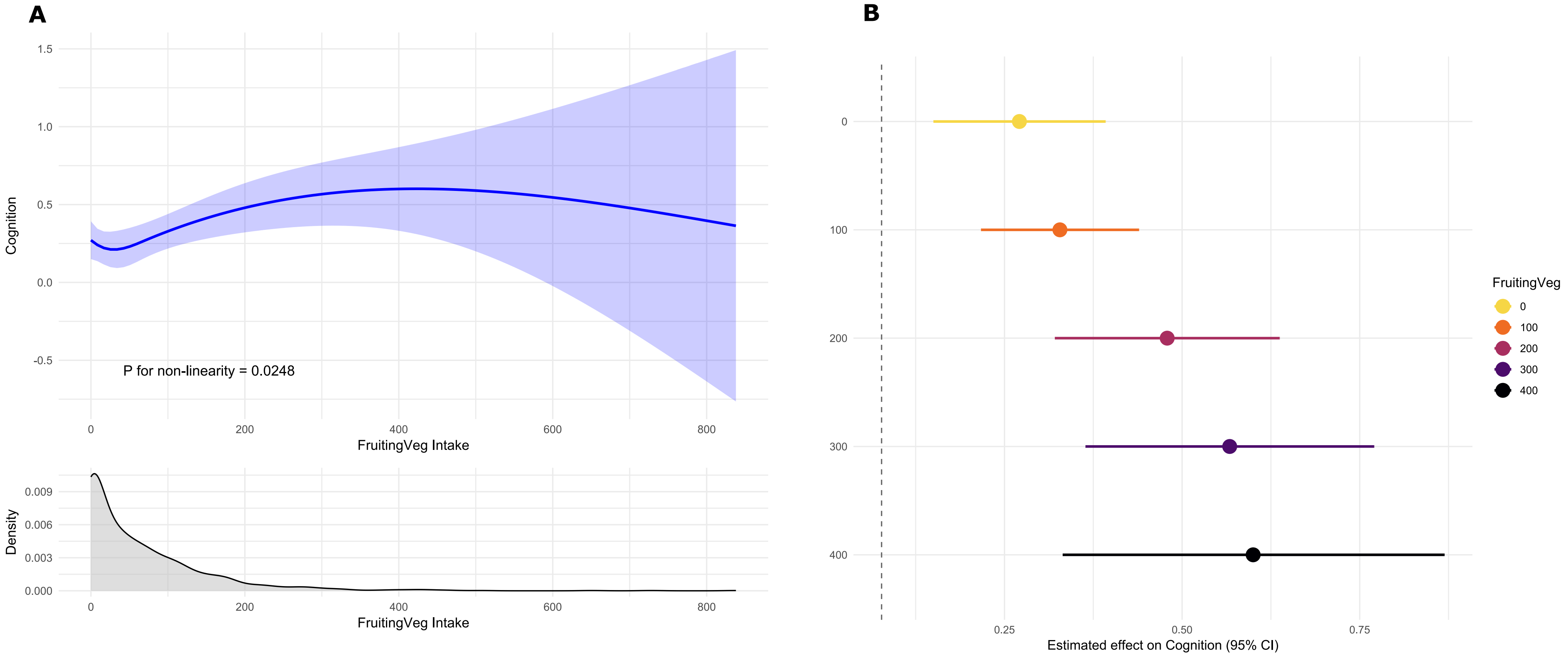

Supplementary figure 4. Association between fruiting vegetables and global cognition.

A. Cubic splines with test for non-linearity; B. Estimated association of several fruiting vegetable intake levels (grams per day) with global cognition composite score.

Both panels present results that are adjusted for gender, age, education, energy intake, leek/onion/garlic group, tea, high fat cheese, other fruit, and berries

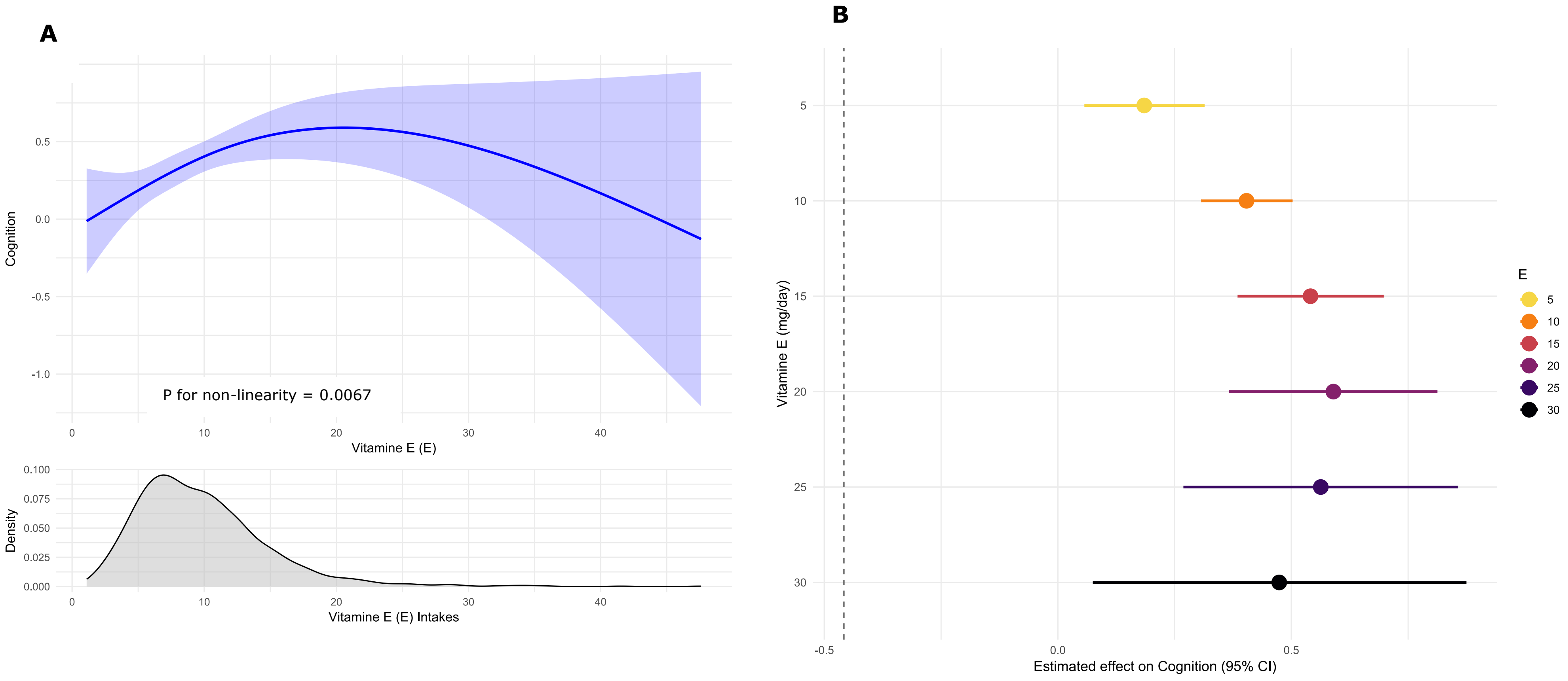

Supplementary figure 5. Association between Vitamin E and global cognition.

A. Cubic splines with test for non-linearity; B. Estimated association of several vitamin E intake levels (milligrams per day) with global cognition composite score.

Both panels present results that are adjusted for age, gender, education, EPA, polysaccharides, cholesterol, vitamin C, omega-6, selenium, beta-carotene, energy intake

Reference

1. Shannon P, Markiel A, Ozier O, Baliga NS, Wang JT, Ramage D, et al. Cytoscape: a software environment for integrated models of biomolecular interaction networks. Genome research. 2003;13(11):2498-504.
